# Enhancing *Pseudomonas syringae* pv. *Actinidiae* sensitivity in kiwifruit by repressing the NBS-LRR genes through miRNA-215-3p and miRNA-29-3p identification

**DOI:** 10.3389/fpls.2024.1403869

**Published:** 2024-07-17

**Authors:** Chengyao Jiang, Xiaoying Zhang, Jiahui Rao, Shu Luo, Liang Luo, Wei Lu, Mengyao Li, Shumei Zhao, Dan Ren, Jiaming Liu, Yu Song, Yangxia Zheng, Yin-Biao Sun

**Affiliations:** ^1^ College of Horticulture, Sichuan Agricultural University, Chengdu, China; ^2^ Laboratory of Crop Immune Gene Editing Technology, Newsun Research Institute of Biotechnology, Chengdu, China; ^3^ Key Laboratory of Agricultural Engineering in Structure and Environment, China Agricultural University, Beijing, China; ^4^ Research Institute of Crop Germplasm Resources, Xinjiang Academy of Agricultural Sciences, Urumqi, China; ^5^ Randall Centre for Cell and Molecular Biophysics, School of Basic & Medical Biosciences, King’s College London, London, United Kingdom

**Keywords:** Kiwifruit bacterial canker, PSA, DEMs and DEGs, miRNA-215-3p and miRNA-29-3p, NBS-LRR

## Abstract

Kiwifruit bacterial canker, caused by *Pseudomonas syringae* pv. *actinidiae* (PSA), poses a grave threat to the global kiwifruit industry. In this study, we examined the role of microRNAs (miRNAs) in kiwifruit’s response to PSA. Kiwifruit seedlings subjected to PSA treatment showed significant changes in both miRNA and gene expression compared to the control group. We identified 364 differentially expressed miRNAs (DEMs) and 7170 differentially expressed genes (DEGs). Further analysis revealed 180 miRNAs negatively regulating 641 mRNAs. Notably, two miRNAs from the miRNA482 family, miRNA-215-3p and miRNA-29-3p, were found to increase kiwifruit’s sensitivity to PSA when overexpressed. These miRNAs were linked to the regulation of NBS-LRR target genes, shedding light on their role in kiwifruit’s defence against PSA. This study offers insights into the miRNA482-NBS-LRR network as a crucial component in enhancing kiwifruit bioresistance to PSA infestation and provides promising candidate genes for further research.

## 1 Introduction

Kiwifruit (*Actinidia* spp.) is a highly nutritious fruit crop widely cultivated worldwide. It is renowned for its richness in vitamin C, dietary fibre, and other essential nutrients, and its unique flavour and health benefits have led to increased popularity in recent years. However, kiwifruit bacterial canker, caused by the gram-negative bacterium *Pseudomonas syringae pv. actinidiae* (PSA), has emerged as a significant threat to the kiwifruit industry (Butler et al., 2013; Kim et al., 2016; Balestra et al., 2018). PSA can result in substantial economic losses due to reduced fruit yield and quality, as well as the expenses associated with control measures (Vanneste, 2017; Li et al., 2021). Additionally, PSA has severe ecological consequences, as it can be dispersed by wind, water, and insects, contributing to the disease’s spread and potential habitat destruction (Accolti, 2015; Spinelli, 2018). First identified in Japan in 1989, PSA has since spread to numerous other countries, including Italy, Chile, New Zealand, and China, among others (Yuichi et al., 1989; Balestra et al., 2011; Scortichini et al., 2012; Vanneste, 2012; Liu et al., 2016). The pathogen can infect all parts of the kiwifruit plant, including leaves, shoots, fruit, and canes, causing symptoms such as leaf wilting, stem cankers, and fruit rot (Donati et al., 2014; Zhou et al., 2015). The disease is highly contagious and can spread rapidly, particularly in conditions of high humidity and temperature. Currently, the primary method for controlling PSA infection relies on the use of copper-based bactericides. However, these can be detrimental to the environment and may lead to the development of copper-resistant strains of the pathogen (Holmes et al., 2014; Pereira et al., 2021). Consequently, the development of resistant cultivars is considered the most cost-effective and environmentally friendly approach to managing this disease.

Plant growth is frequently affected by a variety of pathogenic microorganisms. Unlike vertebrates, plants lack mobile immune cells and an adaptive immune system. They rely primarily on two interrelated layers of the innate immune system to sense and respond to pathogen infections (Jones and Dangl, 2006; Boller and Felix, 2009; Thomma et al., 2011; Spoel and Dong, 2012). One uses cell surface pattern-recognition receptors (PRRs) to recognize microbe-associated molecular patterns (MAMPs) present in a large group of microbes and host-derived damage-associated molecular patterns (DAMPs) (Boller and Felix, 2009), and the other class utilizes disease-resistant (R) proteins to respond to effector molecules secreted by pathogens to help establish a successful infection and suppress plant immunity (Upson et al., 2018). The perception of MAMPs or DAMPs by PRRs activates defence against invading pathogens, termed as pattern-triggered immunity (PTI) (Jones and Dangl, 2006). for successful infection, pathogens have evolved a variety of effectors that are delivered to plant cells to interfere with PTI (Jones and Dangl, 2006; Boller and Felix, 2009). Under such conditions, a plant’s effector-triggered immune (ETI) response is initiated, wherein the pathogen’s effector proteins are recognised and neutralized by proteins encoded by resistance (*R*) genes (Kourelis and van der Hoorn, 2018). These *R* genes typically trigger robust and specific responses, such as the hypersensitive response (HR), which induces cell death at the infection site, thereby impeding pathogen proliferation. Most plant *R* genes encode intracellular nucleotide binding-site leucine-rich repeat (NLR, also known as NBS-LRR) proteins (Han, 2019). Additionally, phytohormones such as salicylic acid (SA), ethylene (ET), jasmonic acid (JA), and abscisic acid (ABA) play crucial roles in enhancing resistance to pathogen-induced stress and extensively utilized by plants (Baldrich et al., 2015).

A pivotal aspect in maintaining plant fitness is the regulation of gene expression via RNA silencing, predominantly mediated by small RNAs (smRNAs). Two main classes of smRNAs are recognized: microRNAs (miRNAs) and short interfering RNAs (siRNAs) (Mallory and Vaucheret, 2006). miRNAs, a subset of small non-coding RNAs, exert significant influence on post-transcriptional gene regulation by binding to complementary sequences on target mRNAs. While considerable research has been dedicated to understanding miRNA responses to abiotic stresses such like drought, cold and salt stress (Yang et al., 2013; Chen et al., 2015; Balyan et al., 2017), comparatively fewer studies have explored miRNA responses to biotic stresses and pathogen infections. Some studies have underscored the importance of miRNAs as essential transcriptional regulators in controlling the expression of various disease-resistant genes. For instance, miRNAs can modulate both the immune response initiated by pathogen-associated molecular patterns and the immune response triggered by effector factors, often containing NBS-LRR-type disease resistance genes (Li et al., 2012), peroxidases, cytochalasin oxidase-encoding genes, *MYB*, and *ARF*, among others., which participate in diverse biological pathways (Li et al., 2010; Zhao et al., 2012).

For example, it has been demonstrated in several plants that the target gene of miR482 is the NBS-LRR gene, influencing plant resistance (Lu et al., 2005; de Vries et al., 2015; Zhang et al., 2016). *Arabidopsis* miRNA393 can be induced by flg22 and negatively regulate plant growth hormones by targeting growth hormone receptor genes *TIR1*, *AFB2*, and *AFB3*, crucial in plant defence against bacterial infestation (Navarro et al., 2006). Additionally, the bacterial flagellin-derived peptide can induce changes in the expression of various miRNAs and their target genes, such as miRNA156, miRNA160, miRNA398, miRNA391, etc. These miRNAs respond to flg22 regulation by modulating the involvement of the target genes in pathways like growth hormone signalling, reactive oxygen species metabolism, and DNA methylation (Li et al., 2010). Ehya et al (Ehya et al., 2013). discovered that ‘*Candidatus phyplasma aurantifolia*’ infestation in Mexican lemon trees led to elevated levels of growth hormones and specific miRNAs (miRNA159, miRNA160, miRNA166) in lemon trees. These miRNAs, along with miRNA156, miRNA166, miRNA167, and their target genes corresponding to ARF and MYB, play pivotal roles in the plant hormone metabolic pathway (Ehya et al., 2013). In a notable study, Zhang et al (Zhang et al., 2016). proposed a coevolution model for plant miRNAs and disease-resistant genes. This model involved the analysis of disease-resistant genes across 70 terrestrial plants combined with extensive miRNA data. Their findings revealed that different miRNAs regulate the expression of disease-resistant genes by targeting conserved structural domains within these genes.

With the continuous advancements in high-throughput sequencing technology and bioinformatics analysis, an increasing number of miRNAs have been discovered in various plants, such as maize, rice, *Arabidopsis*, soybean, grape, apple, and tomato. These miRNAs play widespread roles in the regulation of pathogen infections caused by bacteria, fungi, and viruses (Bazzini et al., 2007; Naqvi et al., 2010; Chen et al., 2012; Yin et al., 2013; Tianzhong, 2014; Wu et al., 2016; Zhou et al., 2016; Yang et al., 2021). Therefore, the identification of miRNAs in kiwifruit in response to PSA and the study of their molecular regulatory mechanisms with target genes hold the potential to provide deeper insights into the molecular mechanisms governing plant-bacteria interactions. This research may also facilitate the development of PSA-resistant kiwifruit varieties.

In this study, we employed high-throughput sequencing technology to examine the expression profiles of miRNAs and mRNAs in kiwifruit seedlings under both normal growth conditions and PSA infection. We conducted a comprehensive genome-wide analysis of the miRNA-mRNA relationships to unveil the specific regulatory mechanisms in kiwifruit during PSA stress, followed by differential expression analysis. Ultimately, we identified two miRNAs that play a role in the response to PSA by regulating NBS-LRR genes. Our study’s findings will contribute to a deeper understanding of the molecular mechanisms governing the interaction between kiwifruit and PSA, and will facilitate the development of PSA-resistant kiwifruit varieties.

## 2 Materials and methods

### 2.1 Materials and plant growth conditions

In this research, we utilized ‘*Hongyang*’ kiwifruit (*Actinidia chinensis*) seeds. These seeds were first washed with 5% sodium hypochlorite for 10 minutes and then rinsed 4-5 times with water. Filter paper moistened with a 500 mg/L gibberellin solution was used to sow the seeds when the moisture content was between 20% and 30%. The soil used for sowing was pre-autoclaved. We employed a permeable seedling tray as a seedbed, spread fine soil evenly on the seedbed, and watered it slowly until the water had penetrated. Subsequently, we sowed the seeds onto the seedbed, covering them with a layer of fine soil approximately 4-6 mm thick. After gently flattening the soil, we covered it with cling film and left it outdoors until the seedlings emerged for subsequent transplanting.

Following transplanting, the kiwifruit seedlings were cultivated in plastic seedling trays (53 × 27.5 × 4.5 cm) filled with substrate (Pindstrup, Denmark) within an artificial climate chamber. The chamber maintained a temperature of 25 ± 1°C during the day and 22 ± 1°C at night, with a relative humidity of 65 ± 5% and a photoperiod of 14 hours. Initially, each group contained 100 seeds. Four weeks after sowing, we selected 60 uniform seedlings, each with two fully expanded leaves, and transplanted them into 7 × 7 × 8 cm black plastic pots filled with substrate (Pindstrup, Denmark). The plants were spaced 10-13 cm apart. For the first four weeks, we irrigated them with a half-dose of Yamazaki nutrient solution (pH 6.5 ± 0.5, EC 1.0 ± 0.2 mS/cm), after which we doubled the dose (EC 2.0 ± 0.5 mS/cm), maintaining this regimen until the end of the experiment.

### 2.2 PSA inoculation

In this study, the PSA strain was isolated from a kiwifruit plantation in Chengdu, China. Referring to the previous method, DNA was extracted and five pairs of specific primers were applied, and the strain was identified as Biovar 3, designated M221 (Andersen et al., 2018), known for its high virulence. To prepare the inoculum, an overnight culture of M221 was introduced into KB liquid medium at a 1% ratio. The culture was shaken at 25°C, 180 revolutions per minute until the OD600 reached a range of 0.4-0.6. Subsequently, the OD600 culture was resuspended using fresh KB liquid medium and adjusted to 0.2. This suspension was drawn up with a sterile 1 ml syringe and grown until the true leaves of the seedlings had fully expanded and exhibited uniform growth. The inoculation involved the injection of leaves from seedlings with fully expanded and uniform growth. Each plant was injected in two leaves, and there were at least 12 plants per treatment. The inoculated plants were placed in a greenhouse with a 12-hour light cycle, daytime temperatures of 25°C, night-time temperatures of 20°C, and humidity levels maintained at 70% or higher. After 48 hours of PSA inoculation, we selected the two true leaves from each plant for miRNA/mRNA-seq analysis.

### 2.3 RNA extraction and illumina sequencing

Total RNAs were extracted from freshly frozen kiwifruit leaves using the MiPure Cell/Tissue miRNA Kit (Vazyme Biotech Co., Ltd, China) for miRNA and the EASYspin Plus Kit (Aidlab Biotechnologies Co. Ltd., Beijing, China) for mRNA, following the manufacturer’s instructions. To assess the quality and quantity of the extracted RNAs, we employed agar gel electrophoresis and a Nanodrop micro spectrophotometer (Thermo Scientific, Wilmington, DE, USA).

For High-throughput sequencing, we combined RNAs from three biological replicates (0.5 g per sample), each derived from at least five plants with the same concentration and volume. We used the MGIEasy Small RNA kit and the NEBNext Ultra RNA library prep kit (NEB#E7530, New England Biolabs, Ipswich, MA, USA) to construct libraries for miRNA and mRNA, respectively. The quality of the cDNA library was assessed using the DNA 1000 assay Kit (5067-1504, Agilent Technologies, Santa Clara, CA, USA) before sequencing. The sequencing was performed on MGI-2000 and Illumina HiSeq TM 2500 platforms by Gene *De novo* Biotechnology Co. (Guangzhou, China). The data were downloaded from the SRA database (accession number: PRJNA1009887 and PRJNA1009946).

### 2.4 Sequencing data analysis

The raw sequencing data were filtered to obtain clean reads for bioinformatics analysis. First, reads with 10% or higher unknown N bases and reads without the 3′adaptor or insert sequences were removed. Then, the 3′ adaptor sequences and reads < 18 nt or > 30 nt were removed. Bowtie (Langmead et al., 2009) was used to BLAST the clean reads against four databases: SILVA, GtRNAdb, Rfam, and Repbase. Candidate miRNAs were obtained by filtering out reads that were identified as ribosomal RNA (rRNA), translocation RNA (tRNA), small nuclear RNA (snRNA), small nucleolar RNA (snoRNA), or repetitive sequences.

### 2.5 Identification of known and novel miRNAs

We used the miRDeep-P2 (1.1.2) (Kuang et al., 2018) to compare reads that were aligned to the kiwifruit reference genome (*Actinidia_chinensis_var.chinensis*) (Wu et al., 2019) with known miRNA precursor sequences in the miRbase database. Reads that were identical to sequences in miRBase were considered to be known miRNAs. Potential miRNA precursor sequences were obtained by aligning the reads to the kiwifruit genome sequence. Reads that did not find matches in miRBase were identified as novel miRNAs by Bayesian model grading based on the location of the reads in the precursor sequence (including mature, star, and loop) and the energy of the precursor structure determined by RNAfold randfold. The reparameterized miRDeep2 is invoked. The length of sequences for predicting RNA secondary structure is set to 250, and a plant-specific scoring system is added to miRDeep2 (Kuang et al., 2018). Although miRDeep2 has been used mainly to identify animal miRNAs, it has been used to identify plant miRNAs after adjusting the parameters and grading system (Zhang et al., 2015).

### 2.6 Differential expression analysis of miRNAs

We calculated miRNA expression level using Transcripts Per Kilobase Million (TPM), which helps mitigate sequencing discrepancy ('t Hoen et al., 2008). The differential expression of miRNA between two sets of samples was analysed by DESeq2 (Wang et al., 2010; Love et al., 2014). We considered miRNAs to be differentially expressed when the log2 fold change ≥1.0 and the p-value was less than 0.001. We calculated TPM and fold change as previously described (Yu et al., 2015).

### 2.7 Prediction of miRNA targets and enrichment analysis

To predict the putative targets of differentially expressed known and novel miRNA candidates, we used PsRobot and TargetFinder software, following the relevant references (Allen et al., 2005; Schwab et al., 2005; Wu et al., 2012). All target genes were subjected to enrichment analysis of Gene Ontology (GO) functions and Kyoto Encyclopedia of Genes and Genomes (KEGG) pathways. In GO enrichment analysis, we identified all GO terms significantly enriched in the target genes compared to the genome background. We mapped all target genes to GO terms in the Gene Ontology database (http://www.geneontology.org/). Significantly enriched GO terms (FDR corrected p-value ≤0.05) were identified using the hypergeometric test, comparing them with the genome background. For pathway enrichment analysis, we utilized the KEGG database. Pathways with FDR-corrected p-values ≤ 0.05 were considered significantly enriched pathways within the target genes.

### 2.8 miRNA-mRNA differential co-expression analysis

We established the association between miRNAs and their target genes by calculating Pearson correlation coefficients using the R package, based on previous research methods (Wang et al., 2019). In general, miRNA-mRNA pairs are negatively correlated because miRNAs function to downregulate their target mRNAs. Pearson correlation coefficients were calculated between each miRNA and its target mRNA. The mRNAs that are significantly inversely correlated with a particular miRNA were selected, and the P value of the correlation coefficient should be less than 0.05, and subsequently re-ran the GO functions and KEGG pathways enrichment analyses.

### 2.9 Expression analysis of miRNAs and predicted target genes using qRT-PCR

We employed stem-loop quantitative reverse transcription PCR (qRT-PCR) to analyse the expression of candidate miRNAs. First, we designed 25 stem-loop reverse transcription (RT) primers and forward primers specific to the selected miRNAs based on their mature sequences. These primers were designed following the instructions provided by the miRNA 1st Strand cDNA Synthesis Kit (Vazyme, China, Nanjing), which can be found at https://www.vazyme.com. For all qRT-PCR reactions, we used universal reverse primers with the sequence AGTGCAGGGTCCGAGGTATT, and *Ac-AcUin* (FG520231) served as the endogenous reference gene.

To validate the expression of miRNAs and target genes through qRT-PCR, we generated first-strand cDNA using the HiScript II 1st Strand cDNA Synthesis Kit (Vazyme, Nanjing, China). The primer sequences for the validation of miRNA and target gene expression are listed in **Supplementary Tables S1**, **S2**, respectively. The qRT-PCR analysis was carried out on a 7900HT Fast Real-Time PCR System (Applied Biosystems, USA) using SYBR qPCR Mix (Invitrogen). The PCR conditions involved an initial step at 95°C for 2 minutes, followed by 40 cycles of 94°C for 20 seconds, 60°C for 20 seconds, and 72°C for 30 seconds. For each qRT-PCR, we ran triplicate technical replicates, and we confirmed amplification products by analysing the melting curve and gel electrophoresis. The relative expression of miRNAs and target genes was calculated using the 2^−ΔΔCt^ method (Livak and Schmittgen, 2001; Jiang et al., 2023).

### 2.10 PCR amplification and plant expression vectors construction

We applied the primers in **Supplementary Table S3** to amplify these five miRNA precursors by PCR, including pre-miRNA-131-3p, pre-miRNA-29-3p, pre-miRNA-107-5p, pre-miRNA-95-3p, and pre-miRNA-215-3p (Precursor coordinates and sequences in **Supplementary Table S4**). The PCR products were gel purified, ligated to pMD18-T vector (Takara), and transformed into competent cells of *Escherichia coli* DH5α (Sambrook and Russell, 2001). Three to five independent clones were selected from each amplicon and used for DNA sequencing (Sangon Biotech, Shanghai, China). Using the In-Fusion^®^ PCR Cloning System (Clontech), the sequenced correct miRNA precursor was cloned into the KpnI site in the pCAMBIA1305-eYGFPuv vector to form the 35S::pre-miRNA-eYGFPuv construct.

### 2.11 Transient expression analysis of five miRNAs

The *Agrobacterium tumefaciens* strain GV3101 carrying the 35S::pre-miRNA-eYGFPuv construct was cultured at 28°C on Luria-Bertani (LB) medium containing the appropriate selection antibiotic. Next, we transferred 500 μL of the culture to a new LB liquid selection medium containing 10 mM 2-(N-morpholino)-ethanesulfonic acid (MES; pH 5.6) and 40 μM acetosyringone and incubated it at 28°C in a shaker. When the bacterial culture reached an OD600 greater than 2.0, we centrifuged the cultures at 4,500 rpm for 10 minutes. The resulting precipitate was resuspended in an infiltration buffer (10 mM MES, 150 μM acetosyringone, 10 mM MgCl_2_), and the bacteria were incubated for 3 hours at room temperature. Subsequently, they were individually injected into 3 mature leaves from a plantlet of annual, uniformly growing kiwifruit, refer to previous methods (Wang et al., 2017; Jiang et al., 2020), using water as a control. The plants were kept at 25 °C for 5 days and inoculated with the PSA pathogens separately, referring to the method 2.2 above. After inoculation, the plants were placed in a greenhouse with a 12-hour light cycle, maintaining a temperature of 25°C during the day and 20°C at night, with humidity levels kept above 70%.

We recorded necrotic areas and disease indices at 8 days post-inoculation (dpi) following established methods (Li et al., 2015). Disease grade (DG) was evaluated according to previous reports (Luan et al., 2016). Subsequently kiwifruit leaves were rinsed with deionized water and immersed in Tepan blue staining solution (consisting of 10 ml deionized water, 10 ml lactic acid, 10 ml glycerol, 10 ml water-saturated phenol, and 10 mg of Tepan blue dye) in a boiling water bath for 1 minute. Afterward, they were placed at room temperature, protected from light, and allowed to stain overnight. The leaves that had been stained overnight were then transferred to 95% ethanol and boiled in a water bath for 10 minutes to remove excess stain. After cooling, they were placed in a new 95% ethanol solution, stored at room temperature, and photographed. Images of the leaves were captured, and spot diameters were measured.

### 2.12 *Agrobacterium tumefaciens*-mediated genetic transformation of kiwifruit

The method of *Agrobacterium tumefaciens*-mediated genetic transformation of kiwifruit was mainly referred to the previous reports (Uematsu et al., 1991; Herath et al., 2020; Yao et al., 2023), and the detailed steps were as follows:

Colonies of *Agrobacterium tumefaciens* GV3101 carrying expression vectors were selected and cultured in LB medium at 28°C and 220 rpm overnight. The organisms were collected by centrifugation at 5000 g for 10 minutes, and then re-suspended in 1/2 MS liquid medium (containing 20 g/L sucrose and 100 µM acetosyringone, pH 5.6), with the OD600 adjusted to 0.5.

New leaves of aseptic kiwifruit seedlings were cut into leaf discs approximately 0.5 cm in size, with the leaf dorsal side facing down. These leaf discs were then cultured on a pre-culture medium MS (containing 2 mg/L 6-BA + 0.2 mg/L NAA + 30 g/L sucrose + 7 g/L agar) in darkness for 3 days. At the end of the pre-culture period, the leaf discs were soaked in an *Agrobacterium infestation* solution for 10 minutes. Subsequently, the bacterial solution was removed by blotting with sterile filter paper, and the leaves were incubated dorsal side down in the dark on a co-culture medium MS (containing 2 mg/L 6-BA + 0.2 mg/L NAA + 100 µM acetosyringone + 30 g/L sucrose + 7 g/L agar) for 2 days.

At the end of the co-culture period, the explants were washed with sterile water, blotted on filter paper, and placed on a screening medium MS (containing 2 mg/L 6-BA + 0.2 mg/L NAA + 250 mg/L Carbenicillin + 10 mg/L Hygromycin B + 30 g/L sucrose + 7 g/L agar) to induce resistant buds. The medium was changed approximately every 20 days during this period. Resistant regeneration buds were obtained after 35 days, and in about 30 days, when the resistant buds grew to about 2-3 cm in size, they were excised and inoculated into a rooting medium 1/2 MS (containing 0.7 mg/L IBA + 250 mg/L Carbenicillin + 10 mg/L Hygromycin B + 30 g/L Sucrose + 7 g/L agar) to induce rooting, and after 2 month, the well-rooted plants were transplanted to the substrate and the genomic DNA of the leaves was extracted. The presence of the transgene in the regenerated plants was further confirmed by PCR using a pair of primers specific for hygromycin (R) and eYGFPuv (**Supplementary Table S3**). qRT-PCR was used to detect the abundance of miRNA-215-3P and miRNA-29-3P in selected positive overexpression lines. 8 months later, 3-6 branches of transgene-positive plants were grafted onto 3-year-old “*Hongyang*” kiwifruit rootstocks for further growth, and one year later, the transgenic plants were analysed for disease resistance.

### 2.13 Resistance analysis of miRNAs transgenic kiwifruit to PSA

Branches of WT and miRNA transgenic kiwifruit plants, with similar thickness, were inoculated with 20 μL of PSA suspension in the phloem of the branches. Three branches from each plant were selected for the injection experiments, and at least six plants were inoculated with each material.

Following inoculation, the plants were placed in a greenhouse with conditions set at 20 ± 1°C and 16 hours of light per day. Necrotic areas and disease indices were recorded at 14 dpi, following established protocols (Li et al., 2015). Disease grade (DG) was evaluated as previously described (Luan et al., 2016).

The abundance of PSA growth was determined by measuring the expression levels of the PSA bacterium *avrE1* gene in kiwifruit leaves using qRT-PCR (Jayaraman et al., 2020). Branch images were taken, and the area of infection was calculated and analysed using the software ImageJ (http://imagej.net). Additionally, leaves were collected to measure the content of malonaldehyde (MDA), as described in previous studies (Li et al., 2015).

## 3 Results

### 3.1 Identification and transcriptome analysis of miRNAs under PSA infection

We first applied M221 to infest red sun kiwifruit leaves to verify its virulence, and found that kiwifruit leaves began to be affected at 2 days of PSA treatment and were most severely affected at 14 days, compared to the control (image not shown). Subsequently, we initiated high-throughput sequencing of kiwifruit leaf samples subjected to PSA infection and control samples. The results indicated that the effective data yield for both groups of samples ranged from 24.50 million to 24.93 million reads. The comparison rate with the kiwifruit reference genome (Wu et al., 2019) was between 73.12% and 80.06%. Reads annotated to the non-coding RNA database accounted for 13.89% to 9.80% of the total, and 442 miRNAs were identified, with lengths mainly in the range of 21-24 bases, with 24 bases being the most common length (**Supplementary Figure S1B**).

A total of 430 miRNAs were identified in all control samples, with 28 of them absent from the PSA samples. In the PSA samples, 414 miRNAs were identified with 12 of them absent from the control samples (**Supplementary Figure S1C**). Among all identified miRNAs, some conserved structural domains were found at positions 18-24 (**Supplementary Figure S1D**).

Further analysis revealed 364 differentially expressed miRNAs (DEMs) with a log2 value of ≥1.5-fold difference and a p-value less than 0.001. These included 251 up-regulated and 113 down-regulated expressions (**Supplementary Table S4**). In parallel, RNA-seq analysis identified 7170 differentially expressed genes (DEGs), comprising 2419 up-regulated and 4751 down-regulated genes (**Supplementary Table S5**).

The miRNA-mRNA differential co-expression analysis unveiled that 133 miRNAs had up-regulated expression, corresponding to 625 target genes with down-regulated expression (**Supplementary Figure S1E**). Additionally, 47 miRNAs exhibited down-regulated expression, corresponding to 16 genes with up-regulated expression (**Supplementary Figure S1E**; **Supplementary Table S6**).

### 3.2 Target gene prediction of DEMs

Using two different software programs, target gene prediction for DEMs revealed that 364 miRNAs corresponded to 31039 target genes (**Supplementary Figure S2A**). Previous studies have reported that resistance genes typically include several highly conserved structural domains, such as the Nucleotide-binding site (NBS) structural domain, Leucine-rich repeat (LRR) structural domain, Toll/Interleukin-1 Receptor-like (TIR), Coiled-coil (CC), and RPW8 (resistance to powdery mildew 8) (Marone et al., 2013; Shao et al., 2016). It’s noteworthy that over 80% of the plant resistance genes identified to date belong to the NBS-LRR protein family (Shao et al., 2016). Additionally, WRKY transcription factors (Jimmy and Babu, 2015; Xu et al., 2015) and E3 ubiquitin ligases (Yu et al., 2013; You et al., 2016) have been frequently associated with plant disease resistance responses, Furthermore, it has been shown that key genes regulating processes such as auxin signal transduction, reactive oxygen species metabolism and DNA methylation, such as the MYB transcription factor and the growth hormone response factor ARF, are highly involved in miRNA-mediated plant-bacteria interactions (Dunoyer et al., 2006; Li et al., 2010).

In this study, we assessed the target genes of DEMs containing the mentioned domains. Our findings showed that among the target genes, 377 contained LRR domains (Involving 319 miRNAs), 167 contained NBS-LRR domains (Involving 322 miRNAs), 9 had NBS domains (Involving 64 miRNAs), 46 contained CC domains (Involving 226 miRNAs), 3 had CC-NBS domains only (Involving 49 miRNAs), 1 contained the Rx_N-NBS domain (Involving 16 miRNAs), 1 contained the RPW8-NBS domain (Involving 15 miRNAs). Additionally, we identified 114 WRKY transcription factors (Involving 246 miRNAs), 673 E3 ubiquitin ligases (Involving 334 miRNAs), 5 genes containing TIR structural domains (Involving 12 miRNAs), 151 MYB transcription factors (Involving 284 miRNAs) and 167 ARF (Involving 271 miRNAs) (**Supplementary Figure S2B**; **Supplementary Table S7**).

### 3.3 GO enrichment analysis of target genes

Performing GO enrichment analysis on the predicted target genes can provide valuable insights into the functions of miRNAs. In the biological process aspect, it was observed that the majority of ;the target genes were associated with cellular processes (8563 genes), metabolic processes (7913 genes), bioregulation (2694 genes), bioprocess regulation (2458 genes), response to stimuli (1659 genes), localization (1544 genes), and organization of cellular components or biogenesis (1488 genes) (**Supplementary Figure S2C**). In the cellular component aspect, most of the target genes were linked to cell membrane components (9146 genes), cell membrane components (8747 genes), cells (7446 genes), and organelles (5962 genes) (**Supplementary Figure S2C**). Regarding the molecular function aspect, a significant number of genes were associated with connectivity (12617) and catalytic activity (11927) (**Supplementary Figure S2C**; **SupplementaryTable S8**).

### 3.4 Determination of miRNAs in response to PSA treatment

**Figure 1:**
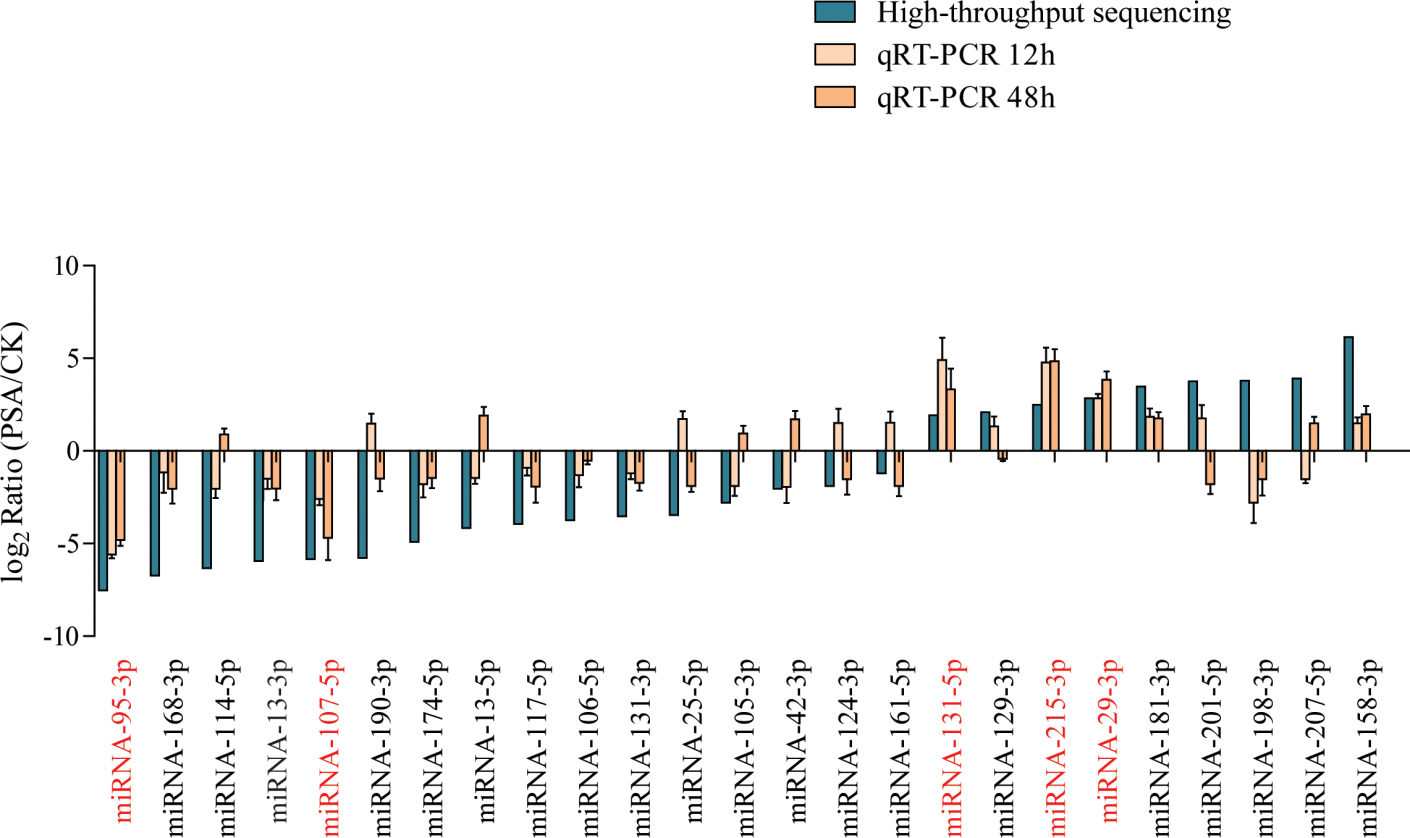
Screening of PSA-responsive miRNAs. Expression levels of 25 miRNAs were examined by qRT-PCR under PSA treatment for 12 h and 48 h. The expression level of each miRNA was calculated relative to that of the respective untreated control samples. Kiwi *Ac*-*AcUin* (*FG520231*) was used as an internal control to normalise the expression data. Different colours represent different treatments. X-axis red markers represent screened candidate PSA-responsive miRNAs and error bars represent standard deviations calculated based on three biological replicates.

We used qRT-PCR to further assess the expression levels of miRNAs under PSA treatment. Considering the miRNA families and their expression patterns, we selected 25 miRNAs, which included 3 miRNA156, 1 miRNA159, 1 miRNA160, 1 miRNA164, 2 miRNA166, 2 miRNA167, 1 miRNA171, 1 miRNA172, 1 miRNA390, 2 miRNA393, 1 miRNA396, 1 miRNA397, 2 miRNA398, 3 miRNA399, 1 miRNA408, and 2 miRNA482 family miRNAs. The results indicated that after 12 and 48 hours of PSA treatment, two miRNAs showed a significant down-regulation in expression, while three miRNAs exhibited a noticeable up-regulated in expression. These findings were consistent with the results obtained through high-throughput sequencing (**Figure 1**).

### 3.5 Functional validation of miRNAs in response to PSA

**Figure 2:**
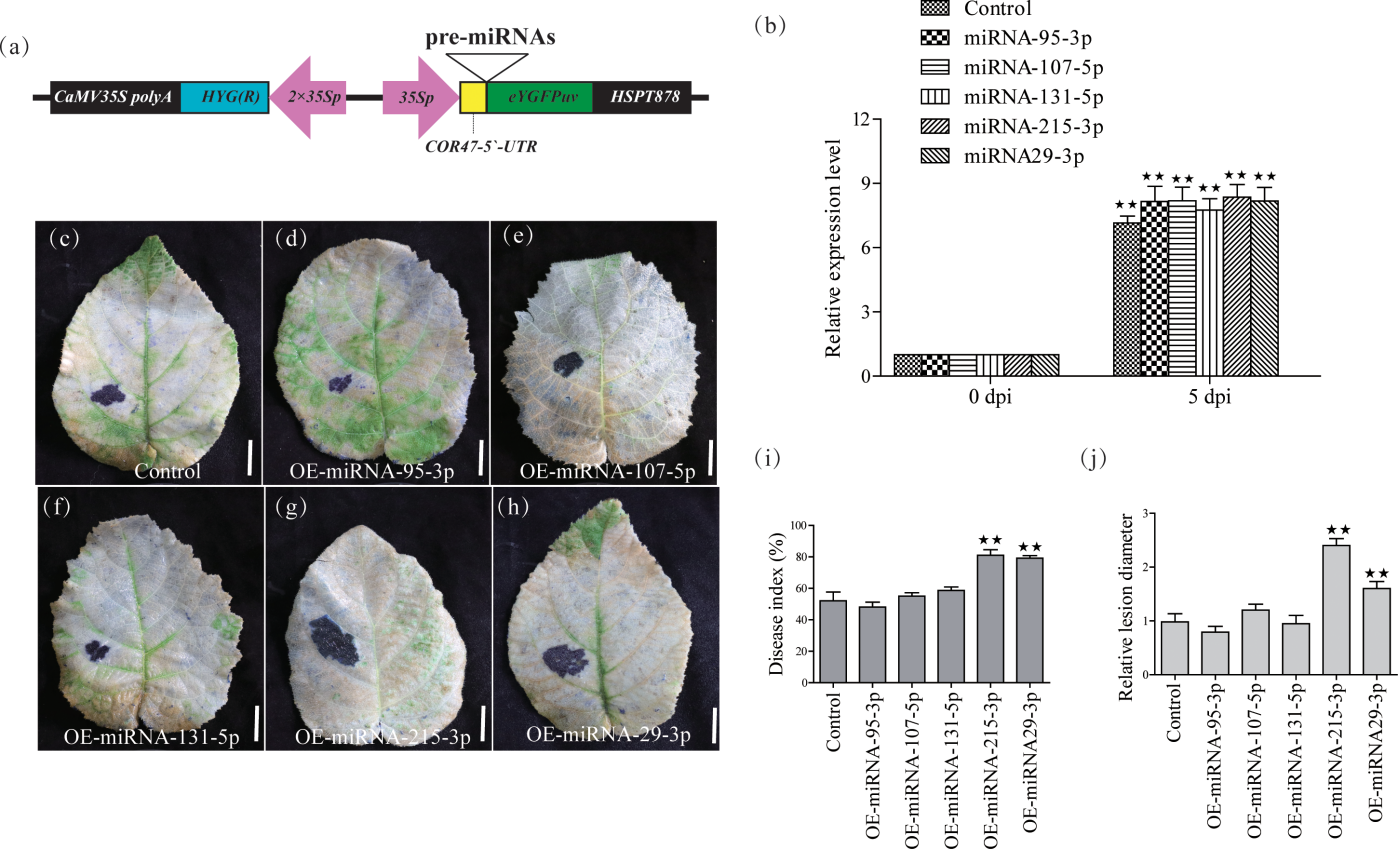
Validation of 5 miRNAs for PSA response functions by transient overexpression analysis in kiwifruit leaves. **(A)** Schematic diagram of the gene cassette used for the overexpression of 5 miRNAs in kiwifruit. **(B)** The relative expression level of the 5 miRNA was calculated relative to the expression in the respective untreated control samples (0 h). Kiwifruit *Ac*-*AcUin* (*FG520231*) was used as an internal control to normalize the expression data. Different colours represent different treatments. The error bars represent the standard deviation calculated based on three biological replicates. **(C–H)** After transient overexpression of the 5 miRNAs in kiwifruit leaves, photographs of plant leaves after placenta blue staining and decolourisation were performed on the 8th day after PSA treatment. Scale bars = 1 cm. **(I)** Disease index (%) at 8 days after PSA inoculation. Control: transient overexpression of empty vector. **(J)** Relative lesion diameter. Data are mean ± SE of three independent experiments. Double asterisks indicate highly significant differences between samples.

To further confirm the functions of these five miRNAs, we constructed five transient overexpression vectors under the control of the 35S promoter (**Figure 2A**). We subsequently transiently overexpressed these vectors in kiwifruit leaves and detected changes in the expression levels of the 5 miRNAs by qRT-PCR, and found that the expression levels of the 5 miRNAs increased 6-10-fold after 5 days (**Figure 2B**), at which time PSA treatment was performed. We evaluated disease indices, relative lesion diameters, and the number of dead cells in kiwifruit leaves on the 8th day after PSA treatment. The results showed that the disease index, relative lesion diameter, and number of dead cells in kiwifruit leaves overexpressing miRNA-215-3p and miRNA-29-3p were significantly higher compared to those in WT plants (**Figures 2C–J**). This indicates that the overexpression of miRNA-215-3p and miRNA-29-3p increased the plant’s susceptibility to PSA. These miRNAs may play a role in the kiwifruit’s response to PSA infection.

### 3.6 Functional identification of miRNA-215-3p and miRNA-29-3p

**Figure 3:**
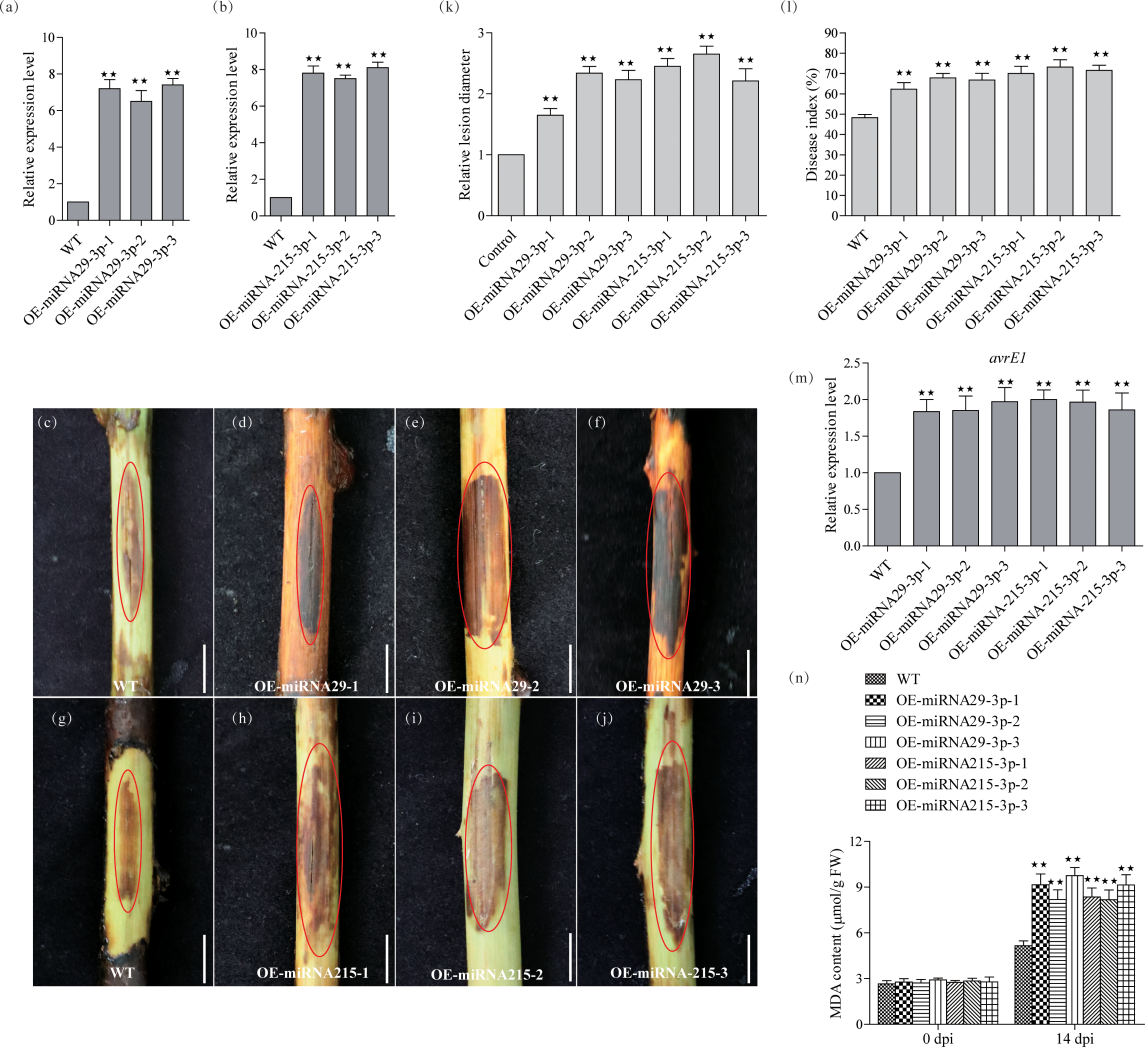
Functional validation of miRNA-29-3p and miRNA-215-3p transgenic kiwifruit against PSA infection. **(A, B)** Quantitative real-time PCR analysis of the abundance of miRNA-29-3p and miRNA-215-3p in WT, miRNA-29-3p overexpressing **(A)**, and miRNA-215-3p overexpressing **(B)** kiwifruit lines. **(C–J)** The stem phloem of 1-year-old transgenic plants was treated with PSA, and after 14 days, the phloem tissue was scraped off to take photographs (scale bar = 1 cm). **(K, L)** Infection site assays on kiwifruit plants 14 days after infection with PSA showed that WT and overexpressing kiwifruit lines had disease indices and relative lesion diameter. **(M)** Transcript accumulation of PSA *avrE1* gene in these inoculated plants 24 h after inoculation with PSA. **(N)** MDA content of the leaves from WT, and overexpressing lines at 14 days after inoculation with PSA. Data are the means ± SEs from three independent experiments. Double asterisks indicate highly significant differences between samples.

To understand the functions of miRNA-215-3p and miRNA-29-3p, we constructed transgenic plants for these two miRNAs separately. Four-week-old kiwifruit seedlings, including both the WT and three representative positive transgenic lines, were transferred to pots and allowed to grow for an additional 2 weeks before genetic testing. After this 2-week growth period, we conducted qRT-PCR analysis to assess the expression levels of miRNA-215-3p and miRNA-29-3p in their leaf tissues. The results showed that miRNA-29-3p was approximately 7.2, 6.9, and 7.4 times more abundant in all overexpression lines compared to WT plants (**Figure 3A**), while miRNA-215-3p was around 7.9, 7.4, and 8.1 times more abundant in all overexpression lines compared to WT plants (**Figure 3B**). Subsequently, after 14 days of PSA treatment, all overexpression plants of miRNA-215-3p and miRNA-29-3p exhibited more severe infections compared to WT plants (**Figures 3C–I**). This observation aligns with the results of the transient overexpression PSA treatments of these two miRNAs (**Figures 2B–I**). Further examination of PSA replication abundance confirmed higher PSA concentrations in all overexpression plants of miRNA-215-3p and miRNA-29-3p after 24h post-inoculation (**Figure 3M**). Additionally, the MDA content was higher in all overexpression plants relative to the WT-shaped plants, indicating severe damage. In summary, these findings suggest that miRNA-215-3p and miRNA-29-3p act as negative regulators of the kiwifruit’s response to PSA.

### 3.7 Identification of candidate target genes

Both miRNA-215-3p and miRNA-29-3p belong to the plant miR482/2118 microRNA superfamily. miRNAs from this family have frequently been linked to plant pathogenicity, with many members known to target NBS-LRR resistance genes, thereby influencing plant resistance (de Vries et al., 2015; Zhang et al., 2016).

**Figure 4:**
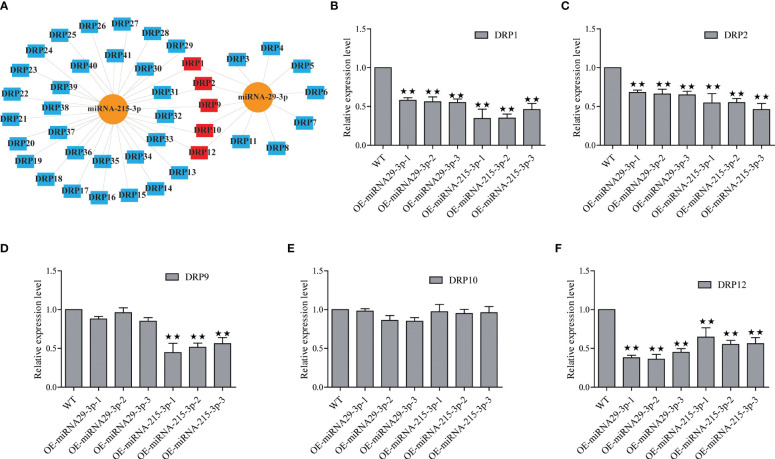
Screening of NBS-LRR target genes (named disease resistance protein, DRP) regulated by miRNA-215-3p and miRNA-29-3p. **(A)** Prediction of DRPs regulated by miRNA-215-3p and miRNA-29-3p, and they regulate 29 and 7 potential DRPs (blue), respectively, and co-regulate 5 DRPs (red). **(B–F)** Expression of 5 DRPs was analysed by qRT-PCR in overexpressing miRNA-215-3p and miRNA-29-3p strains 24 h after inoculation with PSA. The relative expression levels of each DRP were calculated relative to the expression levels of the WT samples. Kiwi *Ac*-*AcUin* (FG520231) was used as an internal control to normalise the expression data. Error bars represent standard deviations calculated based on three biological replicates. Double asterisks indicate highly significant differences between samples.

**Figure 5:**
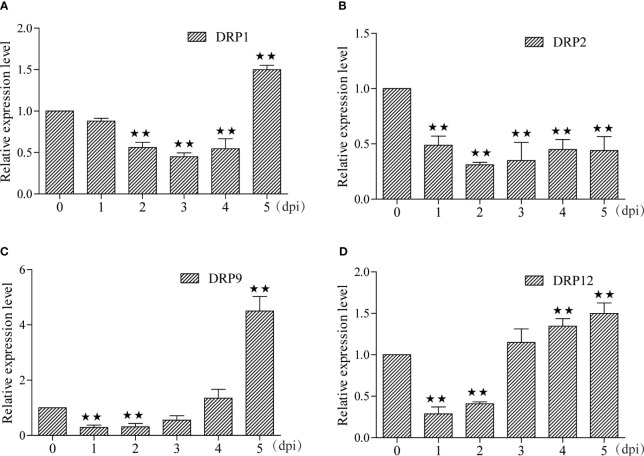
Expression pattern analyse of these four DRPs under PSA treatment. **(A–D)** The expression levels of the four DRPs in kiwifruit leaves were measured by qRT-PCR from 1 to 5 dpi after PSA spraying. Data are means ± SE of three independent experiments; single asterisks indicate significant differences between samples, and double asterisks indicate highly significant differences between samples.

In this study, we identified 29 and 7 potential NBS-LRR target genes designated as DRP (Disease resistance protein), for miRNA-215-3p and miRNA-29-3p, respectively. Intriguingly, we found that these two miRNAs shared 5 common DRPs (**Figure 4A**; **Supplementary Tables S7**, **S9**). Suggesting that miRNA-215-3p and miRNA-29-3p may be involved in multiple pathogenic microbial stress responses through extensive regulation of DRPs, and they may have functional redundancy and co-regulate 5 DRP-like target genes involved in PSA stress response. Firstly, we examined the expression levels of the 5 DRP target genes co-regulated by both of them by qRT-PCR, which revealed significant down-regulation of DRP1, DRP2, and DRP12 in miRNA-215-3p and miRNA-29-3p overexpressed lines. Additionally, DRP9 was down-regulated exclusively in miRNA-215-3p overexpressed plants, while DRP10 remained unaffected (**Figures 4B–F**). Secondly, we analysed the expression patterns of these four DRP genes in response to PSA treatment, and the expression levels of all four DRPs were significantly reduced on the first and second days after PSA treatment (**Figures 5A–D**). This behaviour contrasts with the expression trends of miRNA-215-3p and miRNA-29-3p (**Figure 1**), providing additional evidence supporting the interaction between miRNA-215-3p, miRNA-29-3p, and these 4 target genes.

The results imply that miRNA-215-3p and miRNA-29-3p do have functional redundancy and divergence, and are involved in plant response to PSA pathogens, either jointly or individually, by regulating multiple DRPs. In addition, there should be many more potential DRPs involved in a wide range of pathogenic microbial stress responses to be further explored. The present study provides insights into the critical role of miRNA482-NBS-LRR network in enhancing biotic resistance to PSA infestation in kiwifruit, and provides promising candidate genes for further studies.

## 4 Discussion

Kiwifruit, an economically significant fruit globally, faces a formidable challenge in the form of bacterial canker disease caused by *Pseudomonas syringae* pv. *actinidiae* (PSA). This disease profoundly hinders the sustainable growth of the global kiwi industry. The pathogen was initially identified in the Hayward variety (*A. chinensis* var. *deliciosa*) in Japan in 1989 (Yuichi et al., 1989), and has since surfaced in other countries (Koh, 1994; Mazarei and Mostofipour, 1994; Scortichini, 1994; Ferrante and Scortichini, 2009). Bacterial canker disease, known for its robust transmission capabilities, has progressively evolved into an epidemic, causing symptoms like wood tissue cankers, leaf spots, bud rot, and, in severe cases, plant fatality and orchard devastation. What adds to the gravity of the situation is that virtually all major kiwifruit varieties are susceptible to PSA infection. Notably, the kiwifruit variety ‘*Hort16A*’ (*Actinidia chinensis* var. *chinensis* ‘*Hort16A*’), which garnered considerable attention and investment in the early 21st century, fell prey to PSA susceptibility. Since November 2010, ‘*Hort16A*’ kiwifruit has been grappling with a widespread bacterial canker disease outbreak, inflicting substantial economic losses upon New Zealand in just a few years (Vanneste, 2017). Following extensive exploration of diverse control methods, researchers have concurred that one of the most economically viable measures entails selecting PSA-resistant kiwifruit varieties (Beatrice et al., 2017; Vanneste, 2017). However, there is a dearth of reports concerning resistance-related genes in kiwifruit. Therefore, the identification and development of these genes in kiwifruit assume paramount importance.

The discovery of miRNA represents a pivotal advancement in the field of RNA research, unveiling a regulatory mechanism inherent in non-coding regions. As vital small RNA molecules deeply implicated in post-transcriptional regulation, miRNAs identify target genes through complementary pairing with plant mRNAs. This recognition triggers the degradation of target mRNAs or inhibits gene translation, ultimately suppressing the expression of these target genes (Bartel, 2009). miRNAs actively partake in the orchestration of plant growth, development, hormone signal transduction, and responses to biotic and abiotic stresses.

However, it’s worth noting that current miRNA research predominantly revolves around model crops and staple cereals like *Arabidopsis*, tobacco, rice, wheat, soybean, and maize. Fruit trees, on the other hand, have received comparatively limited attention. Most investigations in the realm of fruit trees have centred on the identification of miRNAs and the prediction of their target genes. Research thus far has indicated that most of the plant and fruit tree miRNAs are evolutionarily conserved, with the identified fruit tree miRNAs often aligning with established microRNA families. Nonetheless, a substantial number of conserved and non-conserved miRNAs, as well as fruit tree-specific miRNAs, remain to be unearthed and characterized. Fruit trees, characterized by protracted growth cycles and intricate genetic mechanisms, present unique challenges in terms of obtaining transgenic plants. Therefore, it becomes imperative to redouble efforts aimed at exploring and comprehending the roles of miRNAs in fruit tree growth, development, and stress resilience.

Despite significant breakthroughs in the study of plant miRNAs, encompassing miRNA identification, target gene prediction, and their roles in plant growth and stress response, there are still several unresolved issues persist (Chen, 2009). These include:

1. The synthesis and regulation of specific miRNAs.
2. The mechanism by which miRNAs select between inhibiting target gene expression or gene cleavage.
3. The limitations of current methods for predicting miRNA target genes based solely on base complementarity.
4. The origin of miRNAs and their significance in biological evolution.
5. The existence of multiple cross-regulatory mechanisms in miRNA-mediated gene regulation.

These challenges have constrained the functional study of plant miRNAs, with only a small fraction having had their functions discovered relative to the total number of miRNAs. In the future, unravelling the regulatory mechanisms of miRNAs on their target genes and identifying the functions of miRNAs involved in plant disease resistance will stand as pivotal themes in miRNA research.

In this study, our focus was on exploring the miRNAs involved in the immune response triggered by the pathogen-associated molecular patterns (PAMPs) of PSA in kiwifruit plants and identifying the target genes related to disease resistance. Firstly, we identified and analysed the miRNAs and transcriptome under PSA infection, resulting in the discovery of 442 miRNAs and 4332 siRNAs. Among these miRNAs, 175 belonged to known miRNA families and 267 were newly identified miRNAs. Differential expression analysis revealed that 364 miRNAs exhibited more than a 2-fold difference in expression between PSA-infected and control samples (**Supplementary Table S4**). RNA-seq analysis, on the other hand, identified 7170 differentially expressed genes (DEGs), comprising 2419 up-regulated and 4751 down-regulated genes (**Supplementary Table S5**). Co-expression analysis of miRNA-mRNA revealed that 180 miRNAs were associated with 641 down-regulated target genes (**Supplementary Table S6**). However, it is important to note that the differentially expressed mRNAs identified through RNA-seq analysis did not always match the predicted miRNA target genes, potentially due to the prediction methods and parameters employed for target gene prediction.

It’s well-established that miRNAs can regulate immune responses triggered by PAMPs of plant pathogens, as well as immune responses initiated by effector proteins. Notably, DRP class disease resistance genes serve as primary targets (Li et al., 2012). For instance, miR159a, miR172a, miR172b, and miR845a have been reported to participate in disease resistance and induce programmed cell death in various plants, including *Arabidopsis* (Mica et al., 2009). Building on this knowledge, Zhang et al. (2016) proposed a co-evolution model of plant miRNAs and disease resistance genes, based on a comprehensive analysis of disease resistance genes and an extensive dataset of miRNA data from 70 land plants, and found that different miRNAs regulate the expression of disease resistance genes through conserved domains (Zhang et al., 2016). To better explore PSA-responsive miRNAs, we classified target genes based on their domains, with a specific focus on NBS-LRR domain genes. As a result, we identified 364 miRNAs that targeted 167 genes containing DRP domains. However, it’s important to note that our RNA-seq data did not detect the expression of many DRP genes, warranting further analysis to understand the underlying reasons for this observation.

Literature reports have shown that miRNAs such as miR393, miR156, miR159, miR160, miR166, miR167, miR391, and miR398 play crucial roles in the plant immune response to bacterial infection (Dunoyer et al., 2006; Navarro et al., 2006; Li et al., 2010). This study represents the first report of miRNAs involved in plant bacterial infection in kiwifruit, as we confirmed the response of five miRNAs to PSA infection using qRT-PCR. However, our analysis was limited to time points at 12- and 48-hours post PSA infection, possibly missing some PSA-responsive miRNAs. Notably, we observed a significant down-regulation of miRNA198-3p following PSA treatment, which contradicted the high-throughput sequencing results. Subsequently, we validated the involvement of two miRNA482 family miRNAs, miRNA-215-3p and miRNA-29-3p, in the kiwifruit immune response to PSA by regulating several DRP resistance genes. These findings lay a robust foundation for dissecting the mechanism of PSA resistance in kiwifruit.

## Data availability statement

The datasets presented in this study can be found in online repositories. The names of the repository/repositories and accession number(s) can be found in the article/**Supplementary Material**.

## Author contributions

CJ: Funding acquisition, Investigation, Project administration, Writing – original draft, Writing – review & editing. XZ: Data curation, Supervision, Writing – original draft. JR: Data curation, Investigation, Writing – original draft. SL: Methodology, Visualization, Writing – original draft. LL: Methodology, Supervision, Writing – original draft. WL: Investigation, Supervision, Writing – review & editing. ML: Methodology, Software, Supervision, Writing – review & editing. YS: Formal analysis, Project administration, Resources, Validation, Writing – review & editing. SZ: Formal analysis, Investigation, Resources, Supervision, Validation, Visualization, Writing – review & editing. DR: Project administration, Resources, Validation, Visualization, Writing – review & editing. JL: Supervision, Validation, Visualization, Writing – original draft, Writing – review & editing. YZ: Formal analysis, Project administration, Resources, Writing – review & editing. YS: Writing – review & editing.

## Supplementary material

Supplementary Figure 1 Identification and characterization of differentially expressed miRNAs (DEMs) and genes (DEGs) between CK and PSA treatment samples. **(A)** Statistical data of the RNA-Seq reads for two groups of 6 samples. **(B)** The distribution of repeat group lengths is plotted. The X-axis represents the different lengths of Clean Reads, and the Y-axis represents the corresponding number of Clean Reads bars. Each colour represents a repeat group, and black line segments inside the bars indicate the maximum and minimum values of the number of copies for reads of that length in samples corresponding to that group. **(C)** A Venn diagram shows DEMs commonly expressed in both CK and PSA treatment samples, as well as those specifically expressed in one but not the other. **(D)** Predicted distribution of nucleotides in miRNAs. Distribution charts of the first nucleotide and each position for miRNAs with different lengths, where larger bars indicate higher proportions. **(E)** Analysis of DEMs, DEGs, and differential co-expression of miRNA-mRNA.

Supplementary Figure 2 Prediction of target genes regulated by DEMs and GO enrichment analysis. **(A)** The number of target genes regulated by DEMs predicted by different software. **(B)** Classification of target genes according to the conserved structural domains in their protein sequences. **(C)** GO enrichment analysis of target genes regulated by DEMs. The x-axis represents the number of genes, and the y-axis represents the GO functional classification. The horizontal coordinate is the number of differentially small RNA target genes (the number is squared for graphical appearance), and the vertical coordinate represents GO terms; there are three categories of GO terms in total, marked with different colours.
