# Supplementary material for: Enhancing Pseudomonas syringae pv. Actinidiae sensitivity in kiwifruit by repressing the NBS-LRR genes through miRNA-215-3p and miRNA-29-3p identification
Source: Front Plant Sci. 2024 Jul 17;15:1403869. doi: 10.3389/fpls.2024.1403869 (PMC11288850; doi:10.3389/fpls.2024.1403869)
Supplement: Supplementary file 1 [file Table_1.docx]

**Table S1**.Stem-loop reverse transcription (RT) primer sequences used in the study.

| **Gene** | **Sequences** | **miRNA Family** |
| --- | --- | --- |
| **For** **stem-loop reverse transcription (RT)** | |  |
| miRNA-106-5p | 5' GTCGTATCCAGTGCAGGGTCCGAGGTATTCGCACTGGATACGACCCTTCC 3' | MIR390 |
| miRNA-13-5p | 5' GTCGTATCCAGTGCAGGGTCCGAGGTATTCGCACTGGATACGACCCACAT 3' | MIR393 |
| miRNA-42-3p | 5' GTCGTATCCAGTGCAGGGTCCGAGGTATTCGCACTGGATACGACAGAGGG 3' | MIR408 |
| miRNA-13-3p | 5' GTCGTATCCAGTGCAGGGTCCGAGGTATTCGCACTGGATACGACCCGACG 3' | MIR393 |
| miRNA-158-3p | 5' GTCGTATCCAGTGCAGGGTCCGAGGTATTCGCACTGGATACGACCTTCCC 3' | MIR167_1 |
| miRNA-181-3p | 5' GTCGTATCCAGTGCAGGGTCCGAGGTATTCGCACTGGATACGACTTCAAC 3' | MIR156 |
| miRNA-198-3p | 5' GTCGTATCCAGTGCAGGGTCCGAGGTATTCGCACTGGATACGACCTTGTC 3' | MIR156 |
| miRNA-25-5p | 5' GTCGTATCCAGTGCAGGGTCCGAGGTATTCGCACTGGATACGACTGGGTT 3' | MIR166 |
| miRNA-207-5p | 5' GTCGTATCCAGTGCAGGGTCCGAGGTATTCGCACTGGATACGACAAAAGA 3' | MIR398 |
| miRNA-131-5p | 5' GTCGTATCCAGTGCAGGGTCCGAGGTATTCGCACTGGATACGACTCAAAG 3' | MIR399 |
| miRNA-161-5p | 5' GTCGTATCCAGTGCAGGGTCCGAGGTATTCGCACTGGATACGACATAGTT 3' | MIR397 |
| miRNA-168-3p | 5' GTCGTATCCAGTGCAGGGTCCGAGGTATTCGCACTGGATACGACGTTGTC 3' | MIR166 |
| miRNA-124-3p | 5' GTCGTATCCAGTGCAGGGTCCGAGGTATTCGCACTGGATACGACTGGAGG 3' | MIR172 |
| miRNA-117-5p | 5' GTCGTATCCAGTGCAGGGTCCGAGGTATTCGCACTGGATACGACAAGAGA 3' | MIR167_1 |
| miRNA-129-3p | 5' GTCGTATCCAGTGCAGGGTCCGAGGTATTCGCACTGGATACGACTGGTGT 3' | MIR399 |
| miRNA-131-3p | 5' GTCGTATCCAGTGCAGGGTCCGAGGTATTCGCACTGGATACGACATTGGC 3' | MIR399 |
| miRNA-174-5p | 5' GTCGTATCCAGTGCAGGGTCCGAGGTATTCGCACTGGATACGACGAAGGT 3' | MIR160 |
| miRNA-114-5p | 5' GTCGTATCCAGTGCAGGGTCCGAGGTATTCGCACTGGATACGACACAGGC 3' | MIR164 |
| miRNA-190-3p | 5' GTCGTATCCAGTGCAGGGTCCGAGGTATTCGCACTGGATACGACGGGAAG 3' | MIR398 |
| miRNA-107-5p | 5' GTCGTATCCAGTGCAGGGTCCGAGGTATTCGCACTGGATACGACATGAAG 3' | MIR396 |
| miRNA-29-3p | 5' GTCGTATCCAGTGCAGGGTCCGAGGTATTCGCACTGGATACGACAGGATA 3' | MIR482 |
| miRNA-201-5p | 5' GTCGTATCCAGTGCAGGGTCCGAGGTATTCGCACTGGATACGACAGACAA 3' | MIR156 |
| miRNA-105-3p | 5' GTCGTATCCAGTGCAGGGTCCGAGGTATTCGCACTGGATACGACGAGAAA 3' | MIR171_1 |
| miRNA-95-3p | 5' GTCGTATCCAGTGCAGGGTCCGAGGTATTCGCACTGGATACGACACCAAA 3' | MIR159 |
| miRNA-215-3p | 5' GTCGTATCCAGTGCAGGGTCCGAGGTATTCGCACTGGATACGACGGTGGG 3' | MIR482 |
