# Supplementary material for: Enhancing Pseudomonas syringae pv. Actinidiae sensitivity in kiwifruit by repressing the NBS-LRR genes through miRNA-215-3p and miRNA-29-3p identification
Source: Front Plant Sci. 2024 Jul 17;15:1403869. doi: 10.3389/fpls.2024.1403869 (PMC11288850; doi:10.3389/fpls.2024.1403869)
Supplement: Supplementary file 2 [file Table_2.docx]

**Table S2**. qRT-PCR primer sequences used in the study.

| **Gene** | **Sequences** | **AGI number** |
| --- | --- | --- |
| **For quantitative RT-PCR** | |  |
| miRNA-106-5pF | 5' GCGCGTATGCTAGTAGCTTTGTT 3' | MIR390 |
| miRNA-106-5pR | 5' AGTGCAGGGTCCGAGGTATT 3' |  |
| miRNA-13-5pF | 5' GCGGATGGAGAATGGTTAATG 3' | MIR393 |
| miRNA-13-5pR | 5' AGTGCAGGGTCCGAGGTATT 3' |  |
| miRNA-42-3pF | 5' CGCGCAAATTCTTCTCCTCTC 3' | MIR408 |
| miRNA-42-3pR | 5' AGTGCAGGGTCCGAGGTATT 3' |  |
| miRNA-13-3pF | 5' GCGCGAGCTTACTGAAAACATT 3' | MIR393 |
| miRNA-13-3pR | 5' AGTGCAGGGTCCGAGGTATT 3' |  |
| miRNA-158-3pF | 5' GCGCAGGGAAAAGTAAGATTT 3' | MIR167 |
| miRNA-158-3pR | 5' AGTGCAGGGTCCGAGGTATT 3' |  |
| miRNA-181-3pF | 5' GCGCACCATAGATGAGGATGTA 3' | MIR156 |
| miRNA-181-3pR | 5' AGTGCAGGGTCCGAGGTATT 3' |  |
| miRNA-198-3pF | 5' GCGAGAAAGAATCCGAAGCA 3' | MIR156 |
| miRNA-198-3pR | 5' AGTGCAGGGTCCGAGGTATT 3' |  |
| miRNA-25-5pF | 5' CGCGGAAAAGTAAAGAGACGAG 3' | MIR166 |
| miRNA-25-5pR | 5' CGCGGAAAAGTAAAGAGACGAG 3' |  |
| miRNA-207-5pF | 5' CGCGCTTCGTGTACTGCT 3' | MIR398 |
| miRNA-207-5pR | 5' AGTGCAGGGTCCGAGGTATT 3' |  |
| miRNA-131-5pF | 5' GCGGTTTTTAACTCCGCCA 3' | MIR399 |
| miRNA-131-5pR | 5' AGTGCAGGGTCCGAGGTATT 3' |  |
| miRNA-161-5pF | 5' GCGTCCGTGCAAAAGGAGA 3' | MIR397 |
| miRNA-161-5pR | 5' AGTGCAGGGTCCGAGGTATT 3' |  |
| miRNA-168-3pF | 5' GCGCTTCATCAAGACCAGG 3' | MIR166 |
| miRNA-168-3pR | 5' AGTGCAGGGTCCGAGGTATT 3' |  |
| miRNA-124-3pF | 5' CGCGAACTCGGTTTCTCATT 3' | MIR172 |
| miRNA-124-3pR | 5' AGTGCAGGGTCCGAGGTATT 3' |  |
| miRNA-117-5pF | 5' CGCCAAGTCCACAAGTACATC 3' | MIR167 |
| miRNA-117-5pR | 5' AGTGCAGGGTCCGAGGTATT 3' |  |
| miRNA-129-3pF | 5' CGCGTACCCTCCAGACTACA 3' | MIR399 |
| miRNA-129-3pR | 5' AGTGCAGGGTCCGAGGTATT 3' |  |
| miRNA-131-3pF | 5' CGCGTCAGATACCTCCACAA 3' | MIR399 |
| miRNA-131-3pR | 5' AGTGCAGGGTCCGAGGTATT 3' |  |
| miRNA-174-5pF | 5' GCGGTTCCTCTGCTTCTTG 3' | MIR160 |
| miRNA-174-5pR | 5' AGTGCAGGGTCCGAGGTATT 3' |  |
| miRNA-114-5pF | 5' CGCGGCCACTTCTTGTATTA 3' | MIR164 |
| miRNA-114-5pR | 5' AGTGCAGGGTCCGAGGTATT 3' |  |
| miRNA-190-3pF | 5' GCGCGTCTTCACCCTTTACTT 3' | MIR398 |
| miRNA-190-3pR | 5' AGTGCAGGGTCCGAGGTATT 3' |  |
| miRNA-107-5pF | 5' GCGGTTGTGGCTTCATCAT 3' | MIR396 |
| miRNA-107-5pR | 5' AGTGCAGGGTCCGAGGTATT 3' |  |
| miRNA-29-3pF | 5' GCGTCTTATCATTTGCCGC 3' | MIR482 |
| miRNA-29-3pR | 5' AGTGCAGGGTCCGAGGTATT 3' |  |
| miRNA-201-5pF | 5' GCGGGTCCTCCGTTTTAAC 3' | MIR156 |
| miRNA-201-5pR | 5' AGTGCAGGGTCCGAGGTATT 3' |  |
| miRNA-105-3pF | 5' GCGATCTCGTTCATTGTGTTCC 3' | MIR171 |
| miRNA-105-3pR | 5' AGTGCAGGGTCCGAGGTATT 3' |  |
| miRNA-95-3pF | 5' CGCGTTGGACCCTGTCTTTC 3' | MIR159 |
| miRNA-95-3pR | 5' AGTGCAGGGTCCGAGGTATT 3' |  |
| miRNA-215-3pF | 5' GCGCGCGCGCCAACCC 3' | MIR482 |
| miRNA-215-3pR | 5' AGTGCAGGGTCCGAGGTATT 3' |  |
| DRP2-RTF | 5' TGACACTGAAGCAAAGAAAAGG 3' | CEY00_Acc03888 |
| DRP2-RTR | 5' TGGGACACTACAGAGATCACAAC 3' |  |
| DRP3-RTF | 5' TGGCCGTCAGTTTGGTTATC 3' | CEY00_Acc03889 |
| DRP3-RTR | 5' TGCCTCTTCTGCACTCTTTTTA 3' |  |
| DRP18-RTF | 5' CCAAGGACGGGAGAAGAACA 3' | CEY00_Acc11042 |
| DRP18-RTR | 5' GTCGCAACGGAGAAAACCAA 3' |  |
| DRP26-RTF | 5' TCATCTTGATTCTCTCTCTTCCC 3' | CEY00_Acc17122 |
| DRP26-RTR | 5' ATAGCTCCTTGCTCCACTCTTAT 3' |  |
| DRP55-RTF | 5' TGGTGCCAAAAAAGAGGGAG 3' | CEY00_Acc33017 |
| DRP55-RTR | 5' TAGATAAATCAGGAACAGCGGT 3' |  |
| *Ac-Actin* F | 5' GCAGGAATCCATGAGACTACC 3' | FG520231 |
| *Ac-Actin* R | 5' GTCTGCGATACCAGGGAACAT 3' |  |
| *Psa_(avrE1)-*F | 5' TGAACAGCATCGACTCGGTTTC 3' | *Pseudomonas syringae* pv. *actinidiae* ICMP 18884 biovar 3 (Psa3) |
| *Psa_(avrE1)-*R | 5' CGAGCAGGCAAACTTTCAGCT 3' |  |
