# Supplementary material for: Enhancing Pseudomonas syringae pv. Actinidiae sensitivity in kiwifruit by repressing the NBS-LRR genes through miRNA-215-3p and miRNA-29-3p identification
Source: Front Plant Sci. 2024 Jul 17;15:1403869. doi: 10.3389/fpls.2024.1403869 (PMC11288850; doi:10.3389/fpls.2024.1403869)
Supplement: Supplementary file 3 [file Table_3.docx]

**Table S3**. pre-miRNA cloning, vector construction and transgenic plant detection primer sequences used in the study.

| **Gene** | **Sequences** | **AGI number** |
| --- | --- | --- |
| **For pre-miRNA cloning** | |  |
| pre-miRNA-131-3pF | 5' GCATATACCAATTCGTTTTCG 3' | MIR399 |
| pre-miRNA-131-3pR | 5' CTATGGTAGAGTTGTTGAATG 3' |  |
| pre-miRNA-29-3pF | 5' TTTTGAGCTTCTGGGAGTTATAG 3' | MIR482 |
| pre-miRNA-29-3pR | 5' CATCAACCCAACTTGGCAAAAA 3' |  |
| pre-miRNA-107-5pF | 5' AAGAGCAAGTCCTGTCATGCT 3' | MIR396 |
| pre-miRNA-107-5pR | 5' TGAATTATTAATTTCACTGAATA 3' |  |
| pre-miRNA-95-3pF | 5' GAGCTTTCTTTAGTTCATTAA 3' | MIR159 |
| pre-miRNA-95-3pR | 5' GGGAGCTCCCTTCAGTCCA 3' |  |
| pre-miRNA-215-3pF | 5' ACAACTCTGTCTCCGCCTATA 3' | MIR482 |
| pre-miRNA-215-3pR | 5' AGGGAGGAGGCACATGAAGC 3' |  |
| **For overexpression vector construction** | |  |
| pre-miRNA-131-3p-Spe1F | 5' GGACAGCCCAGATCAACTAGTTGCACTTCTCCTTTGGCAA 3' | |
| pre-miRNA-131-3p-BamH1R | 5' GCCCTTGCTCACCATGGATCCCAGGGCAATTCTCCTTTGG 3' | |
| pre-miRNA-29-3p-Spe1F | 5' GGACAGCCCAGATCAACTAGTGGAATGGGTAGCATGGGAAG 3' | |
| pre-miRNA-29-3p-BamH1R | 5' GCCCTTGCTCACCATGGATCCTCGGAATGGGCGGCCTTGG 3' | |
| pre-miRNA-107-5p-Spe1F | 5' GGACAGCCCAGATCAACTAGTTTCCACAGCTTTCTTGAACTTC 3' | |
| pre-miRNA-107-5p-BamH1R | 5' GCCCTTGCTCACCATGGATCCTATCCCACAGCATTCTTGAAC 3' | |
| pre-miRNA-95-3p-Spe1F | 5' GGACAGCCCAGATCAACTAGTGAGCTTTCTTTAGTTCATTAA 3' | |
| pre-miRNA-95-3p-BamH1R | 5' GCCCTTGCTCACCATGGATCCGGGAGCTCCCTTCAGTCCA 3' | |
| pre-miRNA-215-3p-Spe1F | 5' GGACAGCCCAGATCAACTAGTACAACTCTGTCTCCGCCTAT 3' | |
| pre-miRNA-215-3p-BamH1R | 5' GCCCTTGCTCACCATGGATCCAGGGAGGAGGCACATGAAG 3' | |
| **For transgenic plant detection** | | |
| hygromycin (R)-F | 5' GTCAGGACATTGTTGGAGCC 3' | |
| hygromycin (R)-R | 5' TTGGGGAGTTTAGCGAGAGC 3' | |
| eYGFPuvF | 5' GGAGAAGTTCGAGTTGGTTGG 3' | |
| eYGFPuvR | 5' TTCAGTTGGAAGGCGTAAGC 3' | |
