# Supplementary figures and images for: Enhancing Pseudomonas syringae pv. Actinidiae sensitivity in kiwifruit by repressing the NBS-LRR genes through miRNA-215-3p and miRNA-29-3p identification
Source: Front Plant Sci. 2024 Jul 17;15:1403869. doi: 10.3389/fpls.2024.1403869 (PMC11288850; doi:10.3389/fpls.2024.1403869)

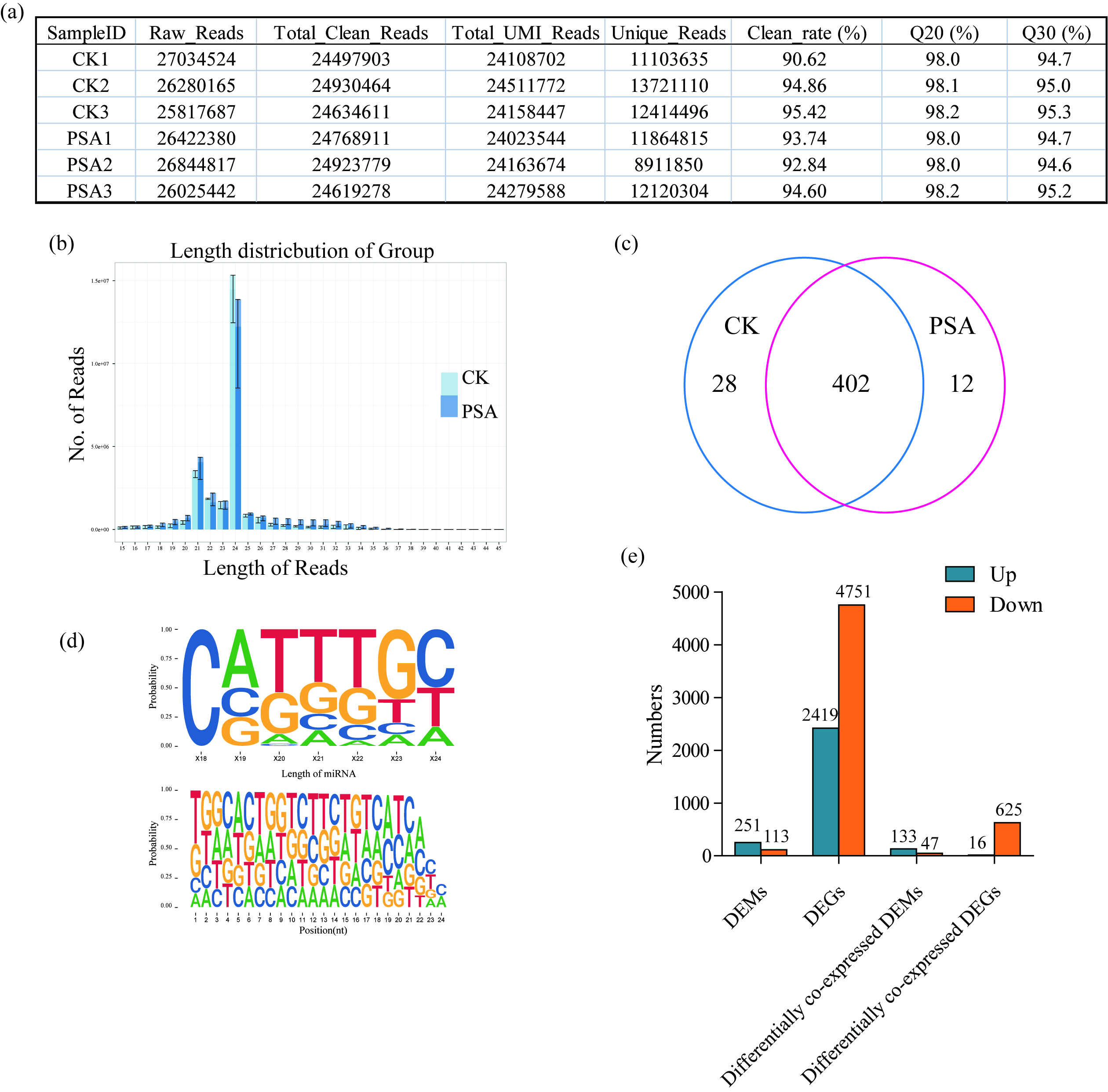

Supplement: Supplementary Figure 1 — Identification and characterization of differentially expressed miRNAs (DEMs) and genes (DEGs) between CK and PSA treatment samples. (A) Statistical data of the RNA-Seq reads for two groups of 6 samples. (B) The distribution of repeat group lengths is plotted. The X-axis represents the different lengths of Clean Reads, and the Y-axis represents the corresponding number of Clean Reads bars. Each colour represents a repeat group, and black line segments inside the bars indicate the maximum and minimum values of the number of copies for reads of that length in samples corresponding to that group. (C) A Venn diagram shows DEMs commonly expressed in both CK and PSA treatment samples, as well as those specifically expressed in one but not the other. (D) Predicted distribution of nucleotides in miRNAs. Distribution charts of the first nucleotide and each position for miRNAs with different lengths, where larger bars indicate higher proportions. (E) Analysis of DEMs, DEGs, and differential co-expression of miRNA-mRNA. [file Image_1.tif]

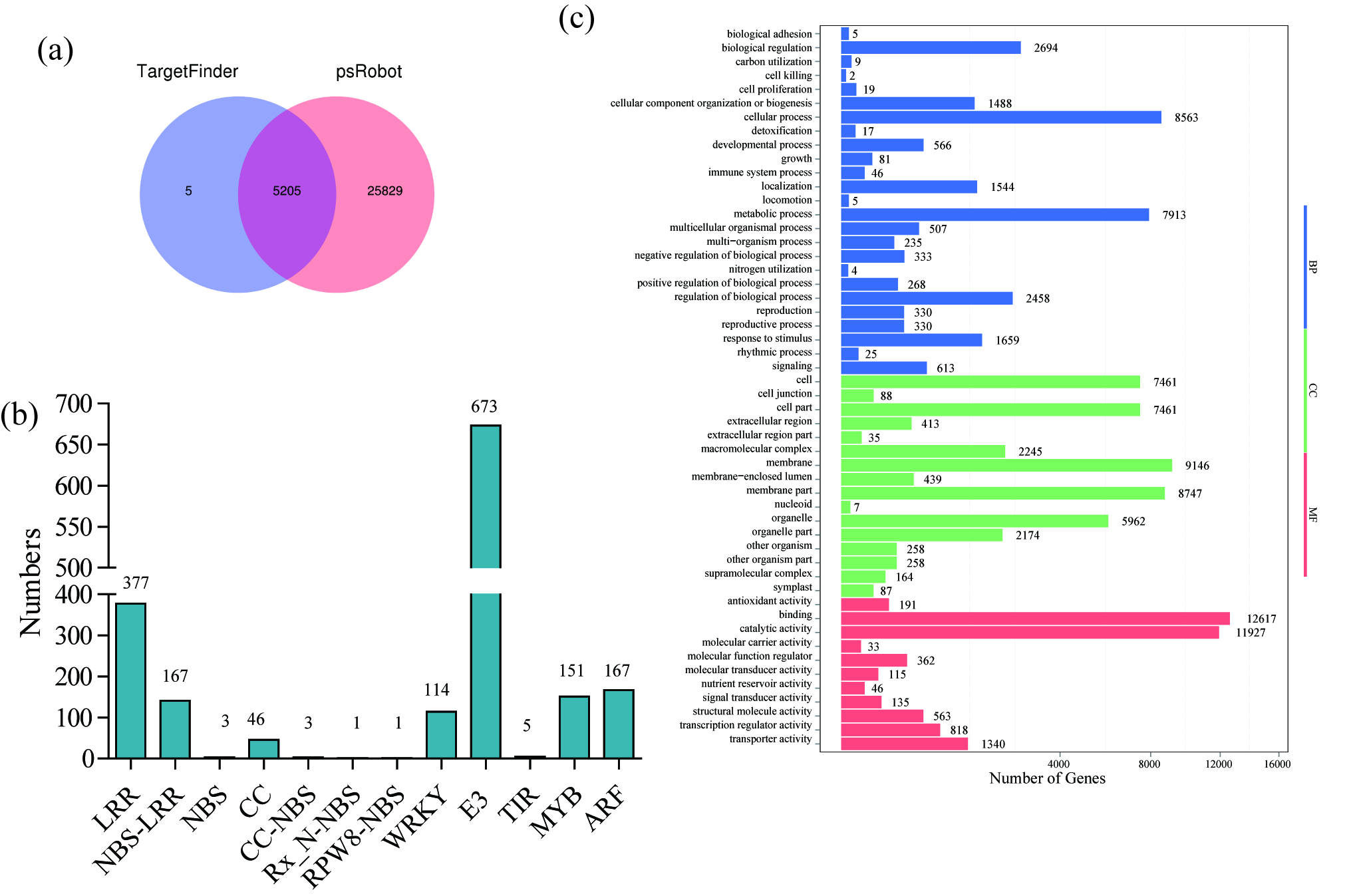

Supplement: Supplementary Figure 2 — Prediction of target genes regulated by DEMs and GO enrichment analysis. (A) The number of target genes regulated by DEMs predicted by different software. (B) Classification of target genes according to the conserved structural domains in their protein sequences. (C) GO enrichment analysis of target genes regulated by DEMs. The x-axis represents the number of genes, and the y-axis represents the GO functional classification. The horizontal coordinate is the number of differentially small RNA target genes (the number is squared for graphical appearance), and the vertical coordinate represents GO terms; there are three categories of GO terms in total, marked with different colours. [file Image_2.tif]
